# Additive-Enhanced SnO_2_ FBG Sensor with Optimized Annealing Time, Temperature, and Multilayer Coating for High-Performance Humidity Sensing

**DOI:** 10.3390/nano15191508

**Published:** 2025-10-01

**Authors:** Soo Ping Kok, Yun Ii Go, Siti Barirah Ahmad Anas, M. L. Dennis Wong, Kah Yoong Chan

**Affiliations:** 1 School of Engineering and Physical Sciences, Heriot-Watt University Malaysia, Putrajaya 62200, Malaysia; k.ping@hw.ac.uk; 2 Wireless and Photonics Networks Research Centre, Faculty of Engineering, Universiti Putra Malaysia, Serdang 43400, Malaysia; 3 School of Engineering, Newcastle University, Newcastle upon Tyne NE1 7RU, UK; 4 Newcastle University Medicine Malaysia, Educity, Johor Bahru 79200, Malaysia; 5 Faculty of Artificial Intelligence and Engineering, Multimedia University, Cyberjaya 63100, Malaysia; 6 Centre for Advanced Devices and Systems, Centres of Excellence for Robotics and Sensing Technologies, Multimedia University, Cyberjaya 63100, Malaysia

**Keywords:** nanostructures, reactive material, sensitivity, precision, adsorption

## Abstract

Coating plays an important role in advancing sensing technology by significantly enhancing sensitivity, stability, and response time. The unique properties of nanostructures, including high surface-to-volume ratio and tunable porosity, make them suitable candidates for improving sensor performance. By optimizing nanostructure coatings, advancements in high-precision humidity sensing devices are achievable, enabling a wide range of industrial applications, especially in humidity-controlled industries. In this study, the effects of annealing time, annealing temperature, and the number of coating layers on the properties of additive-enhanced SnO_2_ nanostructure were investigated. The experiment was carried out by subjecting the additive-enhanced SnO_2_ nanostructure to different annealing times and annealing temperatures to analyze its impact on crystallinity, porosity, and moisture adsorption properties. Upon optimizing the annealing parameters, multilayer coatings were carried out to assess the effect of the total number of coating layers on hygroscopic behavior. A hygroscopicity test was carried out on each sample to evaluate its moisture adsorption and desorption capabilities. The results demonstrated that controlled annealing conditions significantly improve the nanostructure’s hygroscopic properties, and the optimized coating layers further enhanced the moisture retention, making the developed SnO_2_ nanostructure a promising candidate for advanced sensing applications.

## 1. Introduction

Metal oxides have gained increasing popularity in sensing advancements across various applications, such as gas sensing, chemical sensing, and humidity sensing [1,2]. Titanium dioxide (TiO_2_)-based nanostructures are commonly used for applications in chemical and gas sensing due to their chemical and thermal stability [3]. One of the optical fiber sensors, known as a fiber Bragg grating (FBG), has been explored with TiO_2_ coating for chemical detection [4]. The results showed an improved sensing capability of more than 10% with the TiO_2_ coating.

Zinc oxide (ZnO) and SnO_2_ have been explored for humidity sensing purposes as hygroscopic nanomaterials, where ZnO exhibits high thermal and chemical stability, and SnO_2_ demonstrates improved surface area and increased oxygen vacancies, providing more active sites for water retention [5]. A ZnO/SnO_2_ composite humidity sensor has been developed via solvothermal synthesis. Results showed that a high-response sensor with improved linearity and stability can be achieved using the metal oxide composite. The designed sensor also showed small hysteresis and short response and recovery times in a relative humidity range of 11% to 95%.

Other metal oxides, including cerium oxide (CeO_2_), iron oxide (Fe_2_O_3_), chromium oxide (Cr_2_O_3_), vanadium oxide (V_2_O_5_), and silicone dioxide (SiO_2_), have also been explored for sensing purposes as summarize in Table 1. The tunable crystallite size and surface morphology, excellent electrical and optical properties, and high thermal stability of metal oxides make them suitable candidates for deployment under harsh and extreme conditions [6,7]. Research has been carried out to improve the hygroscopicity of the nanomaterial for its applications in humidity sensors with enhanced sensitivity, response time, selectivity, and stability [8].

An additive that acts as a surfactant and structure-directing agent is one of the approaches for the modification of pore structure, resulting in increased hygroscopicity of metal oxide sensing materials [16,17]. An additive-enhanced nanostructure requires controlled annealing and coating processes to achieve the desired structural parameter, particle size, and surface-to-volume ratio. The annealing process is important in optimizing the porosity, surface area, morphology, optical properties, and adsorption capacity of nanostructures [18,19].

The annealing time and temperature are critical parameters to achieve high crystallinity, ensuring the performance and stability of the nanostructure [20]. The unwanted residue and impurities cannot be fully removed under insufficient annealing, jeopardizing the formation of M–O bonds [21]. Whereas over annealing could lead to decomposition of the additive and incur agglomeration, which leads to a reduction in surface area [22].

A multilayer coating increases the thickness of the nanostructure, resulting in a larger particle size [23]. Additionally, this approach produces abundant functional groups and pore structure, providing more active sites for water molecules. However, the repeatability and stability of multilayer nanostructures can be impacted by the interlayer interaction at high humidity conditions [24].

Hybrid layer coating showed improvement in increased surface area, enhanced interfacial bonding for stronger adhesion through the anchoring effect, and improved charge transfer by pairing p-type and n-type semiconductor materials to form a p–n heterojunction [25,26,27].

Optical fiber sensors have been deployed for multiple applications owing to their advantages, including small form-factor, high robustness towards hazardous environments, and ease of integration into wearable smart sensing devices [28,29]. By applying suitable coating materials and optimizing coating techniques to optical fiber sensors, including FBG, improved performance in humidity sensing can be achieved.

To the best of our knowledge, additive-enhanced SnO_2_ nanostructures have not been studied for application in optical-based humidity sensing. The coating parameters of the nanostructure, such as annealing time, annealing temperature, and multilayer coating, have not been analyzed.

In this study, an additive-enhanced SnO_2_ nanostructure will be synthesized, and the effect of annealing time and temperature on the nanostructure will be examined. Multilayer coating of SnO_2_-HMT and hybrid layer coating of ZnO/SnO_2_-HMT will also be investigated. The sensing performance of the synthesized nanostructure will be studied with a hygroscopicity test and characterization.

## 2. Materials and Methods

### 2.1. Additive-Enhanced SnO_2_ Synthesis

**Figure 1:**
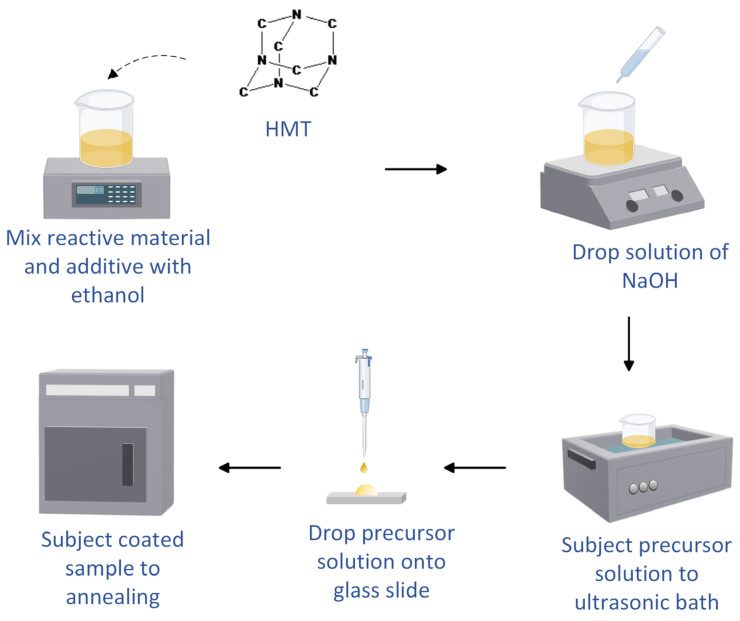
Schematic diagram of low-temperature hydrothermal synthesis.

The SnO_2_-HMT was synthesized via a modified low-temperature hydrothermal technique as illustrated in Figure 1, where tin (II) chloride dihydrate (SnCl_2_∙2H_2_O) (Merck KGaA, Darmstadt, Germany) was selected as the precursor and hexamethyltriethylethanolamine (HMT) (Merck KGaA, Darmstadt, Germany) was dispersed as the additive. 0.01M of precursor and 0.01M of additive were dissolved with 95% ethanol and facilitated by the solution of sodium hydroxide (NaOH) for further dissolution to form a homogeneous coating solution.

### 2.2. Investigation of Annealing Time and Temperature

Glass slides cleaned with 95% ethanol were placed on a hotplate heated up to 80 °C. 100 µL of SnO_2_-HMT coating solution was drop-cast onto the glass slide and let dry for 1 min. The same steps were repeated 5 times to ensure a total of 500 µL precursor solution was deposited to fully cover the substrate for single-layer coating.

The coated glass slide will be subjected to an annealing oven preheated to 140 °C. The annealing time was adjusted to 2.5 h for sample curing. The sample was removed upon completion of the annealing process, followed by the insertion of the sample into the preheated oven with the annealing time set to 3 h and 3.5 h, respectively, to obtain the second and third batches of the sample.

The three samples of fully cured nanostructures were subjected to a hygroscopicity test, where the samples were inserted into a humidity-controlled chamber separately for mass change measurement under increasing and decreasing RH. A hygroscopicity test was conducted, and characterization, including FTIR, microscope image, and FESEM, was carried out. Upon analysis of the testing and characterization results, the optimal annealing time was determined for the next experiment.

The same coating steps will be repeated to obtain another 3 batches of samples; each of the samples will be inserted into the annealing oven separately, with the annealing temperature set to 100 °C, 140 °C, and 180 °C, respectively. The temperature ranges were selected due to the decomposition temperature of HMT is approximately 236.55 °C [30]. Therefore, the annealing temperature in this experiment should not exceed 200 °C to prevent decomposition of the additive. Upon annealing, all samples underwent the same testing and characterization procedure to analyze the effects of annealing temperature on the humidity sensing properties of SnO_2_-HMT.

### 2.3. Investigation of Multilayer and Hybrid Layer Coating

For multilayer coating, the coating steps were repeated to first form a single-layer coating of SnO_2_-HMT on the glass substrate. The sample was subjected to an annealing process with the optimal annealing time and temperature, determined by the hygroscopicity test for first-layer curing. The annealing process was repeated with the same parameters until 3 layers coating were formed.

To obtain a hybrid layer-coated sample, Zinc Oxide (ZnO) was selected as the interlayer between the substrate and the SnO_2_-HMT. The ZnO coating solution was prepared with similar steps by replacing the precursor with zinc acetate dihydrate (Zn(CH_3_CO_2_)_2_∙2H_2_O) (Merck). The sample was prepared by coating and annealing a single-layer ZnO, followed by the coating and annealing of SnO_2_-HMT as the top layer.

Both the multilayer SnO_2_-HMT and hybrid layer ZnO/SnO_2_-HMT samples were subjected to a hygroscopicity test, followed by characterization to examine the water–surface interaction between the nanostructure with the surrounding water molecules.

## 3. Results and Discussions

### 3.1. Hygroscopicity Test

The hygroscopicity test was carried out according to the ASTM standard [31], where the samples were placed within a humidity-controlled chamber and the mass change over increasing RH from 40 to 80%, and decreasing RH from 80 to 40%, was recorded at every 10%RH interval.

**Figure A1:**
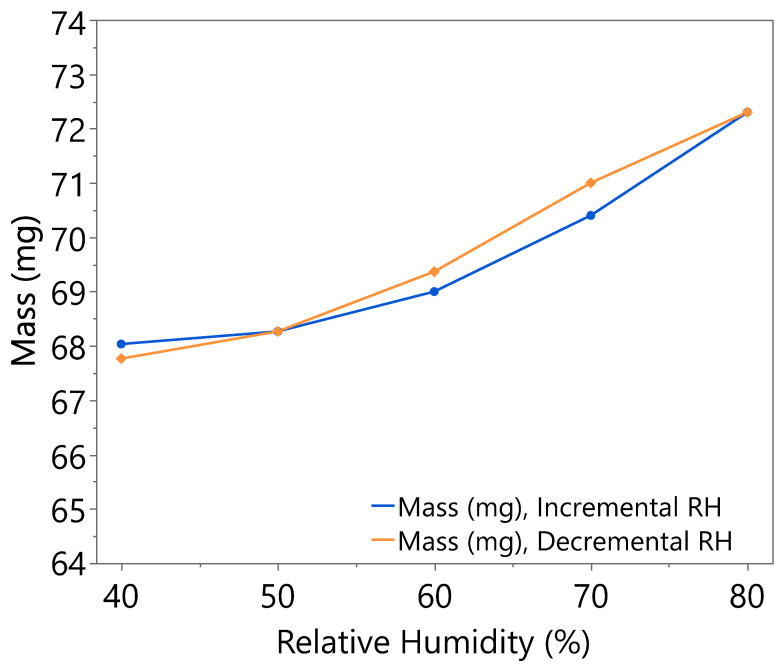
Hygroscopicity test result of 2.5 h annealing time, illustrated as material mass change with incremental and decremental RH.

**Figure A2:**
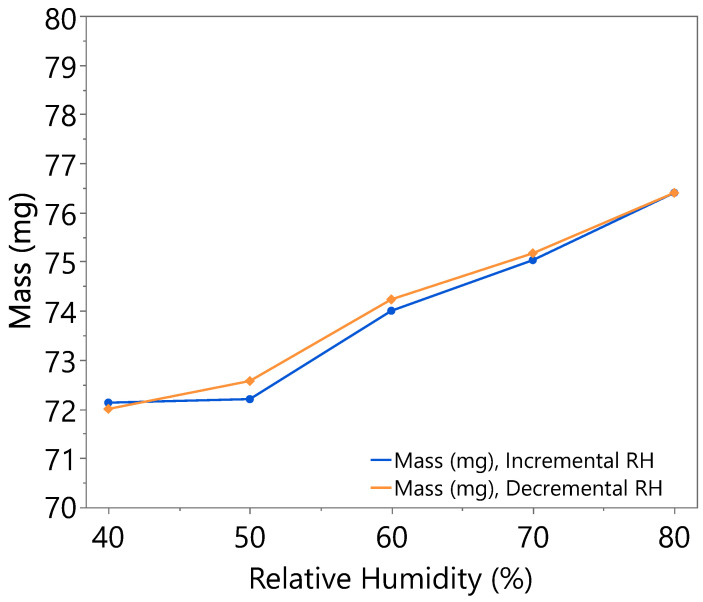
Hygroscopicity test result of 3.0 h annealing time, illustrated as material mass change with incremental and decremental RH.

**Figure A3:**
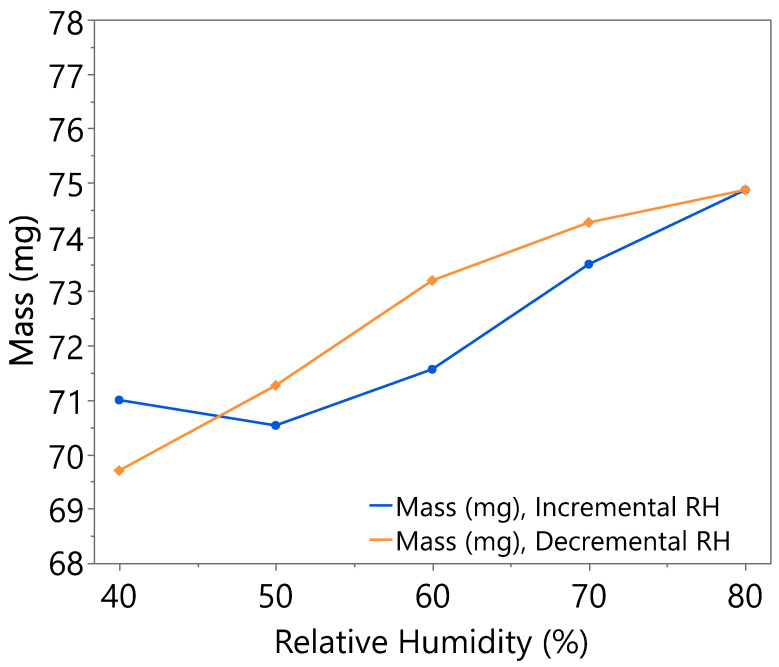
Hygroscopicity test result of 3.5 h annealing time, illustrated as material mass change with incremental and decremental RH.

**Figure A4:**
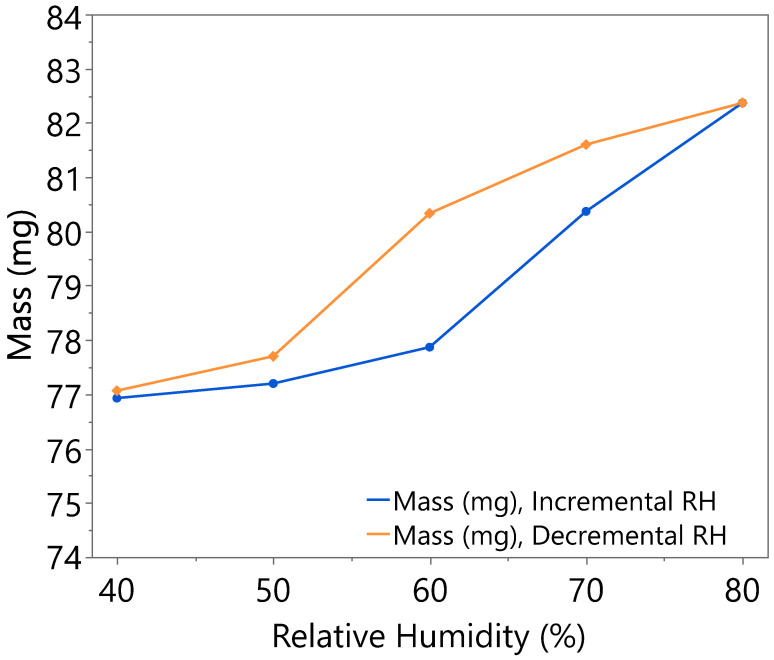
Hygroscopicity test result of 100 °C annealing temperature, illustrated as material mass change with incremental and decremental RH.

**Figure A5:**
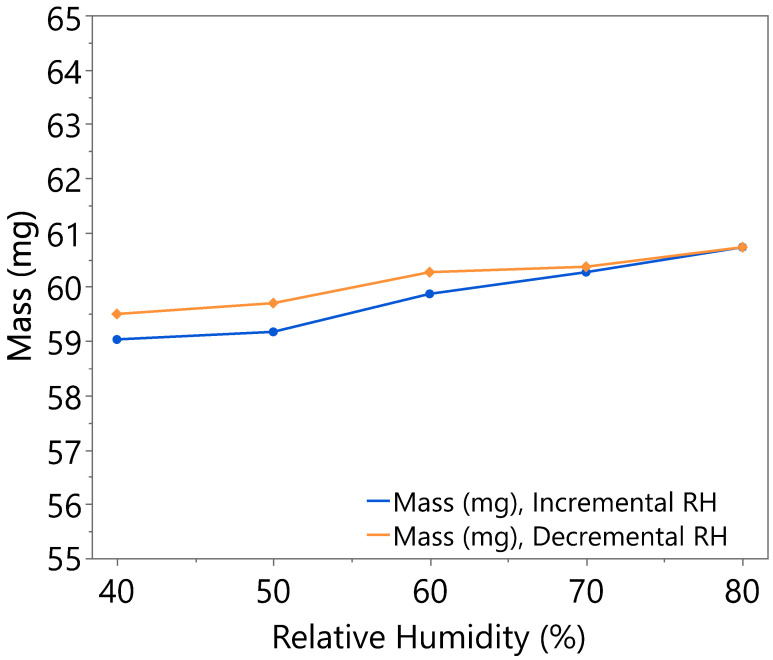
Hygroscopicity test result of 180 °C annealing temperature, illustrated as material mass change with incremental and decremental RH.

**Figure A6:**
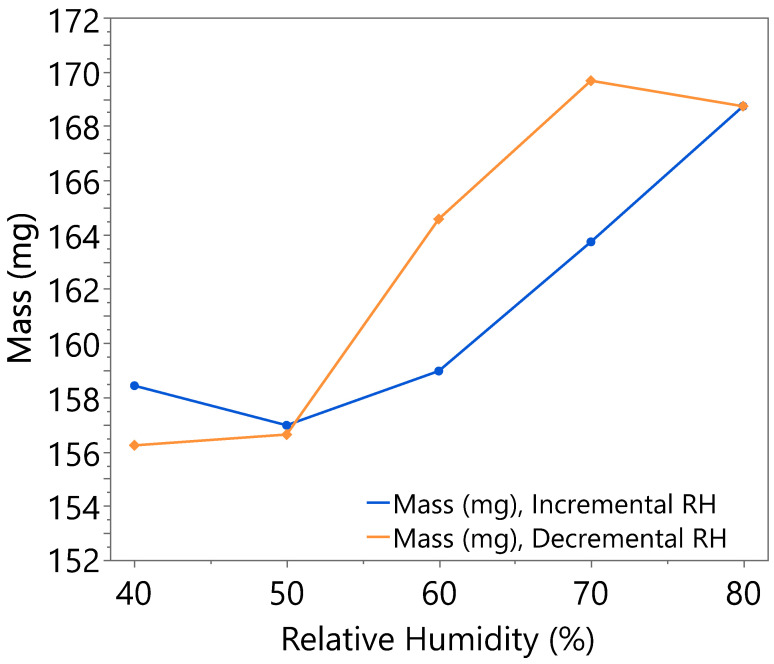
Hygroscopicity test result of 3 layers SnO_2_-HMT, illustrated as material mass change with incremental and decremental RH.

**Figure A7:**
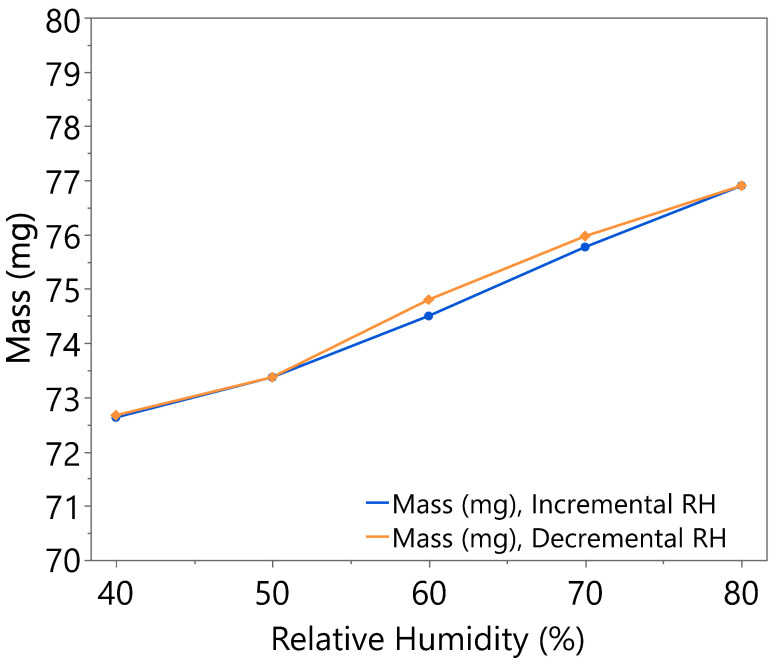
Hygroscopicity test result of hybrid layers ZnO/SnO_2_-HMT, illustrated as material mass change with incremental and decremental RH.

The mass uptake ratio was calculated based on the recorded individual material mass, as illustrated in Figure A1, Figure A2, Figure A3, Figure A4, Figure A5, Figure A6 and Figure A7, by reference to the oven-dried material mass. This ratio quantifies the capability of the nanomaterial in water absorption, adsorption, and desorption. From the linear equation of y = mx + c, the *m value* represents the slope of the line. A higher *m value* indicates higher mass change throughout the humidity cycle, demonstrating better hygroscopicity properties of the tested material.

**Figure 2:**
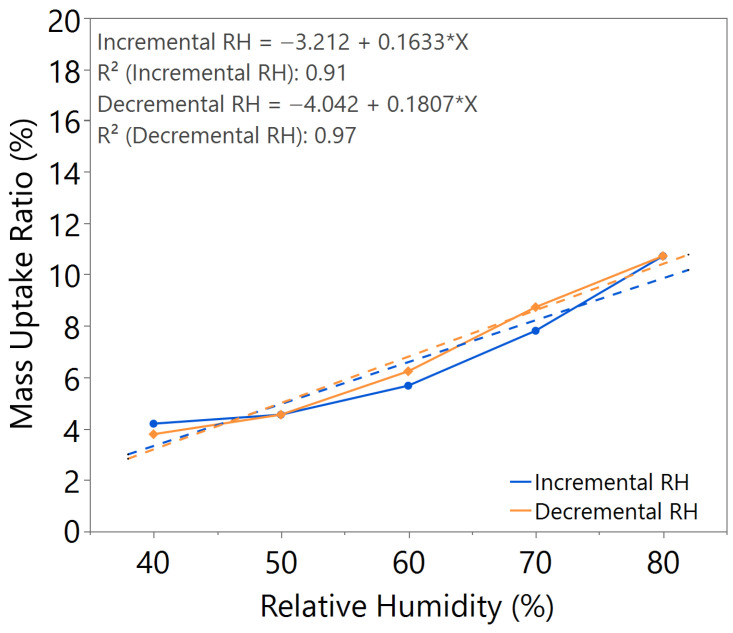
Hygroscopicity test result of 2.5 h annealing time, illustrated as mass uptake ratio with incremental and decremental RH.

**Figure 3:**
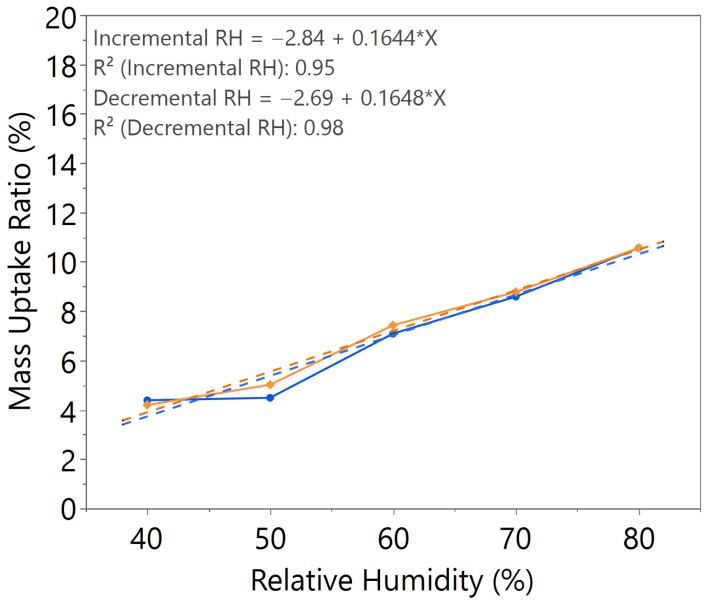
Hygroscopicity test result of 3.0 h annealing time, illustrated as mass uptake ratio with incremental and decremental RH.

Comparing samples with 2.5 h and 3 h annealing time under the same annealing temperature, as illustrated in Figure 2 and Figure 3, both samples exhibited comparable *m value*. However, the sample with 2.5 h annealing time shows slightly higher hysteresis compared to the sample with 3 h annealing time, especially at high humidity levels between 60 and 80%RH, where the material mass is unable to return to its original state during decreasing humidity from 80 to 50%RH.

**Figure 4:**
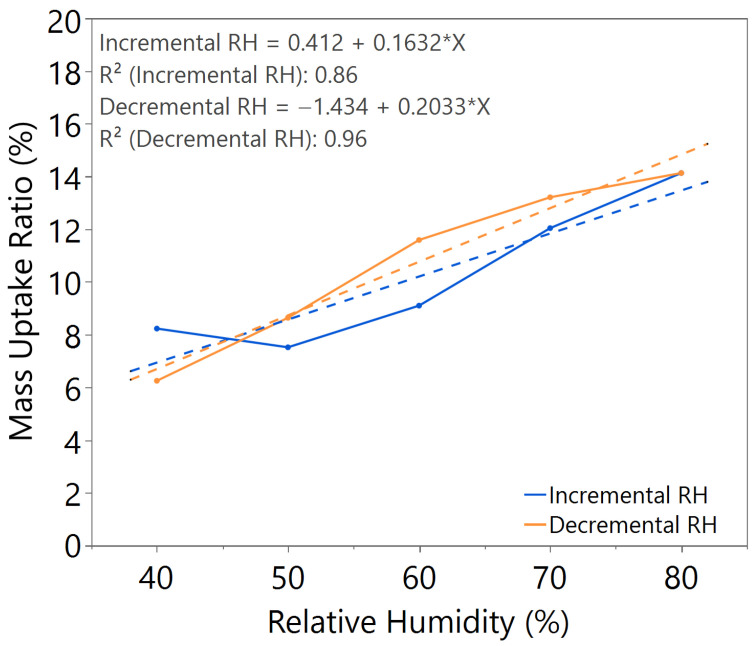
Hygroscopicity test result of 3.5 h annealing time, illustrated as mass uptake ratio with incremental and decremental RH.

**Figure 5:**
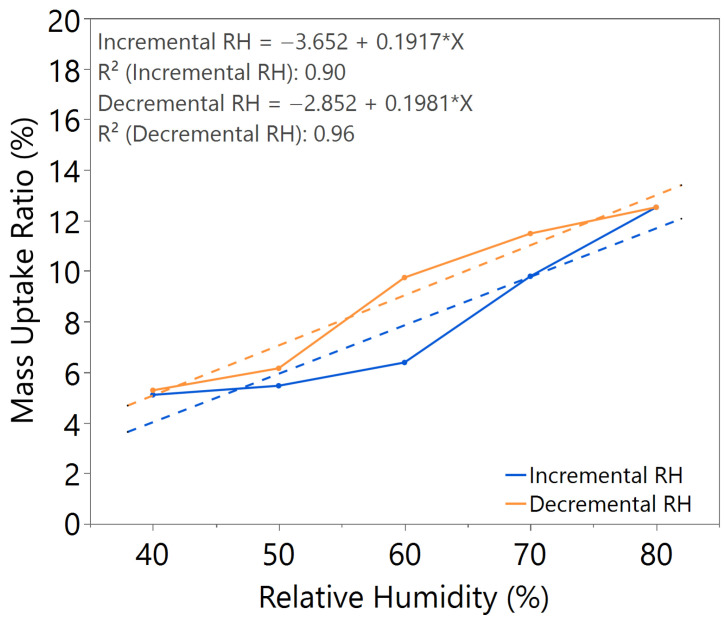
Hygroscopicity test result of 100 °C annealing temperature, illustrated as mass uptake ratio with incremental and decremental RH.

**Figure 6:**
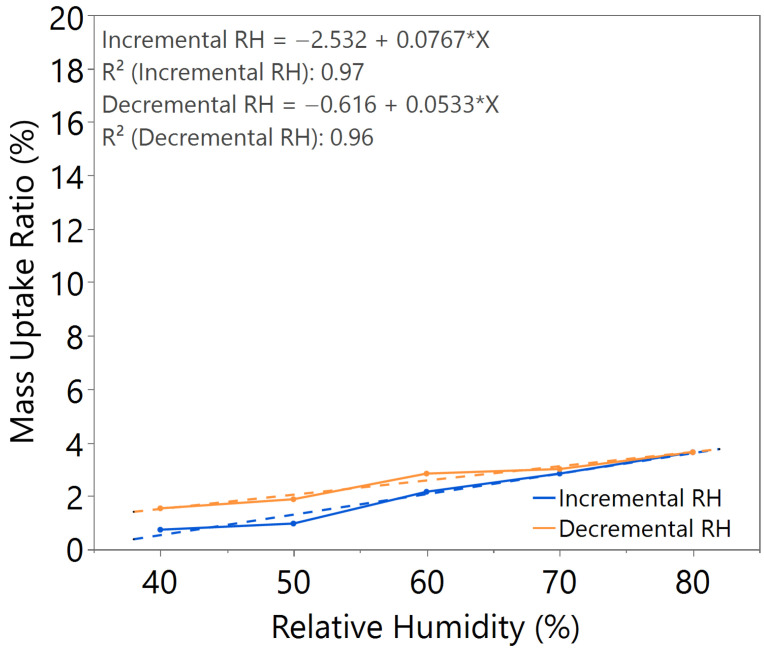
Hygroscopicity test result of 180 °C annealing temperature, illustrated as mass uptake ratio with incremental and decremental RH.

The sample with a 3.5 h annealing time in Figure 4 showed slightly better *m value*, but significant hysteresis was observed. Similar behavior was observed in the sample with a 3 h annealing time under 100 °C annealing temperature, as shown in Figure 5, where a higher *m value* led to higher hysteresis. For the sample with a 3 h annealing time under 180 °C annealing temperature, as illustrated in Figure 6, the mass uptake ratio is low, indicating the lowest water uptake with increasing RH. The sample also showed increased hysteresis throughout the humidity cycle.

From the comparative study of all the samples, a 3 h annealing time with 140 °C annealing temperature showed the most significant water–surface interaction, with an *m value* of approximately 0.16. The samples exhibited negligible hysteresis, indicating that an optimal annealing time and temperature led to the formation of a nanostructure with improved humidity sensing properties, as illustrated in Table 2. 

**Figure 7:**
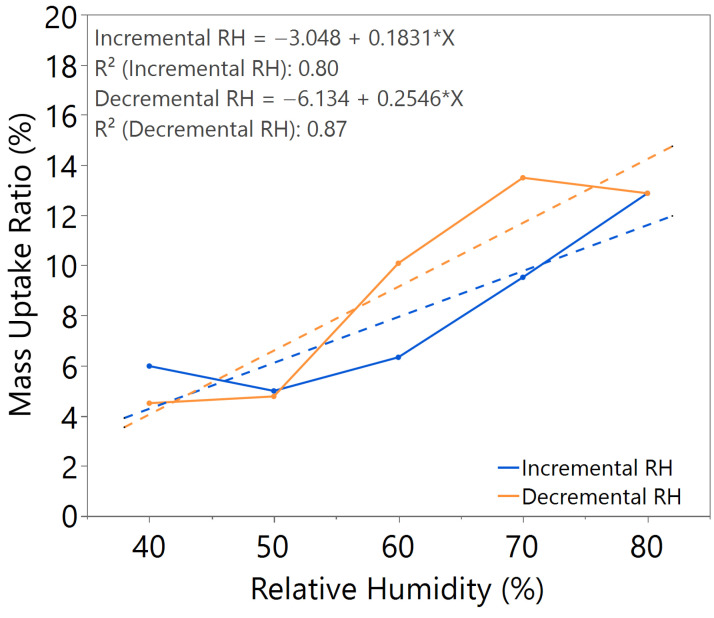
Hygroscopicity test result of 3 layers SnO_2_-HMT, illustrated as mass uptake ratio with incremental and decremental RH.

**Figure 8:**
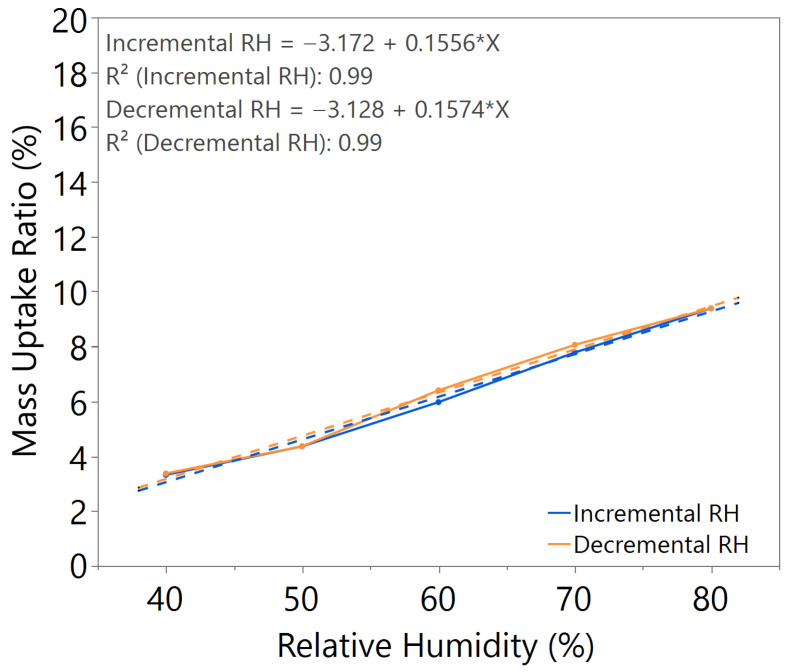
Hygroscopicity test result of hybrid layers ZnO/SnO_2_-HMT, illustrated as mass uptake ratio with incremental and decremental RH.

The samples with a 3-layer coating were synthesized based on the optimal annealing time and temperature. From the hygroscopicity test results, as shown in Figure 7, a significant increase in *m value* and hysteresis was observed. Whereas for hybrid layer coating, the *m value* and hysteresis were comparable with those of single-layer SnO_2_-HMT under 3 h 140 °C annealing conditions, as demonstrated in Figure 8, indicating the ZnO interlayer has minimal impact on the humidity sensing properties of the top coating layer. This could be due to ZnO not affecting the surface morphology of the top-layer nanostructure. Therefore, both hybrid layer coating and single layer coating shared similar porous structures, which contribute to improved water adsorption and desorption mechanisms, leading to minimal hysteresis.

### 3.2. Fourier Transform Infrared Spectroscopy (FTIR)

The FTIR, ranging from 4000 to 420 cm^−1^, identified the O-H stretching, interaction between HMT and SnO_2_, and Sn-O stretching vibrations. Broad peak in the range of 3000–3800 cm^−1^ is attributed to the stretching of hydrogen-bonded O-H groups from moisture adsorption on the surface of synthesized nanostructures. Strong peaks within the range of 400–700 cm^−1^ are attributed to the Sn–O stretching vibrations and the O–Sn–O bending vibrations of SnO_2_-HMT. Whereas peaks at ~820 cm^−1^, ~1000 cm^−1^, ~1250 cm^−1^, and ~1670 cm^−1^ correspond to the out-of-plane C-N stretching, O-H bending, and C-O stretching vibration of the functional groups of HMT, indicating interaction between HMT and SnO_2_.

**Figure 9:**
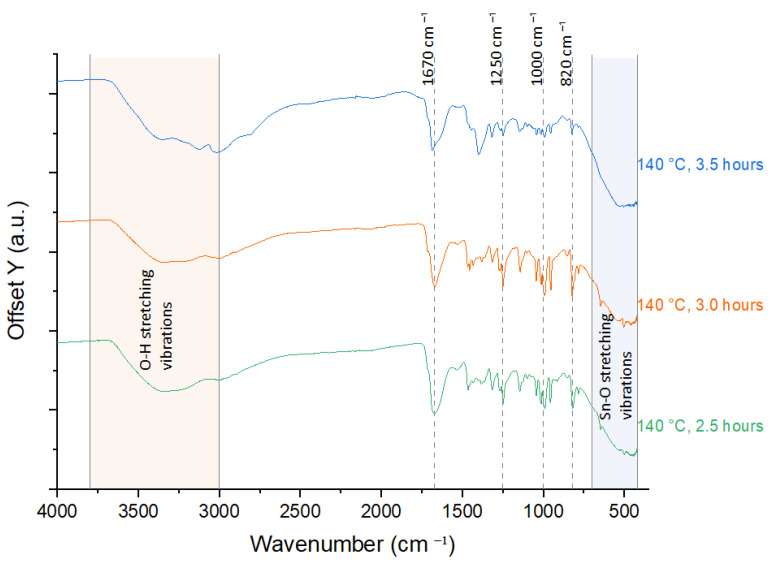
FTIR spectra of 2.5 h, 3 h, and 3.5 h annealing time.

**Figure 10:**
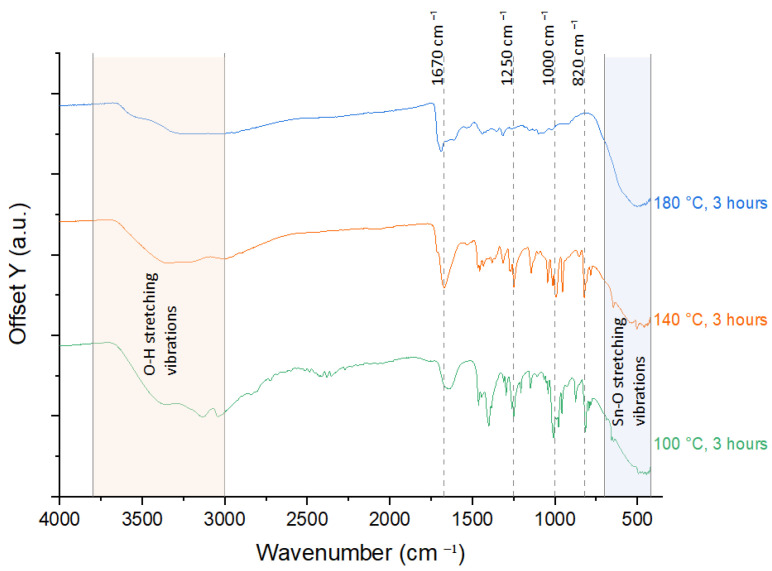
FTIR spectra of 100 °C, 140 °C, and 180 °C annealing temperature.

From the comparative study in Figure 9 and Figure 10, all samples showed strong O-H stretching with a broad peak observed from 3000 to 3800 cm^−1^, except for the sample annealed at 180 °C for 3 h, which showed a rather flat peak in this range. The broad O-H stretching peak represents the presence of hydrogen bonding interactions between the samples and water [32]. The flat peak in the sample annealed at 180 °C for 3 h validated the results observed from the hygroscopicity test, where high annealing temperature led to poor hydrogen bonding interaction, resulting in minimal water uptake throughout the humidity cycle.

The sample went through 140 °C for 3.5 h annealing and 180 °C for 3 h, observed minor peaks at ~820 cm^−1^, ~1000 cm^−1^, ~1250 cm^−1^, and ~1670 cm^−1^, which can be due to decomposition of HMT under over-annealing conditions. The strongest peaks at these locations were observed in the sample with 100 °C, 3 h annealing, which can be attributed to unreacted HMT that remained on the sample surface. This result indicated that optimal annealing conditions are important for HMT to react with sensing materials for structural modifications.

### 3.3. Microscope Image

**Figure 11:**
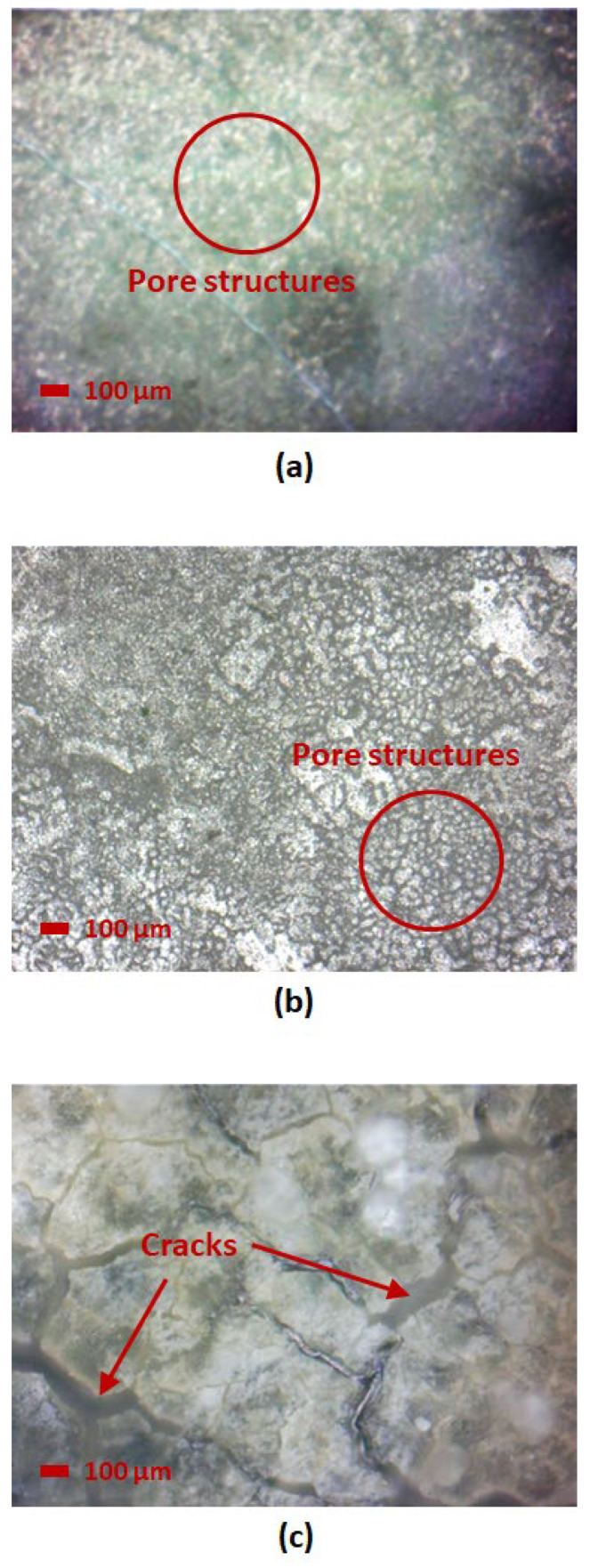
Microscope image at 20× magnification of (**a**) 2.5 h, (**b**) 3 h, and (**c**) 3.5 h annealing time.

Microscope images of each sample were obtained under 20× magnification to observe the structural change of SnO_2_-HMT under different annealing and coating conditions. A comparison of different annealing times is shown in Figure 11, where annealing times of 2.5 h and 3 h both led to the formation of pore structures. No pore structure was observed after an annealing time of 3.5 h, which verified the result obtained from FTIR, indicating that the missing pores can be attributed to the decomposition of HMT at high annealing time and temperatures.

**Figure 12:**
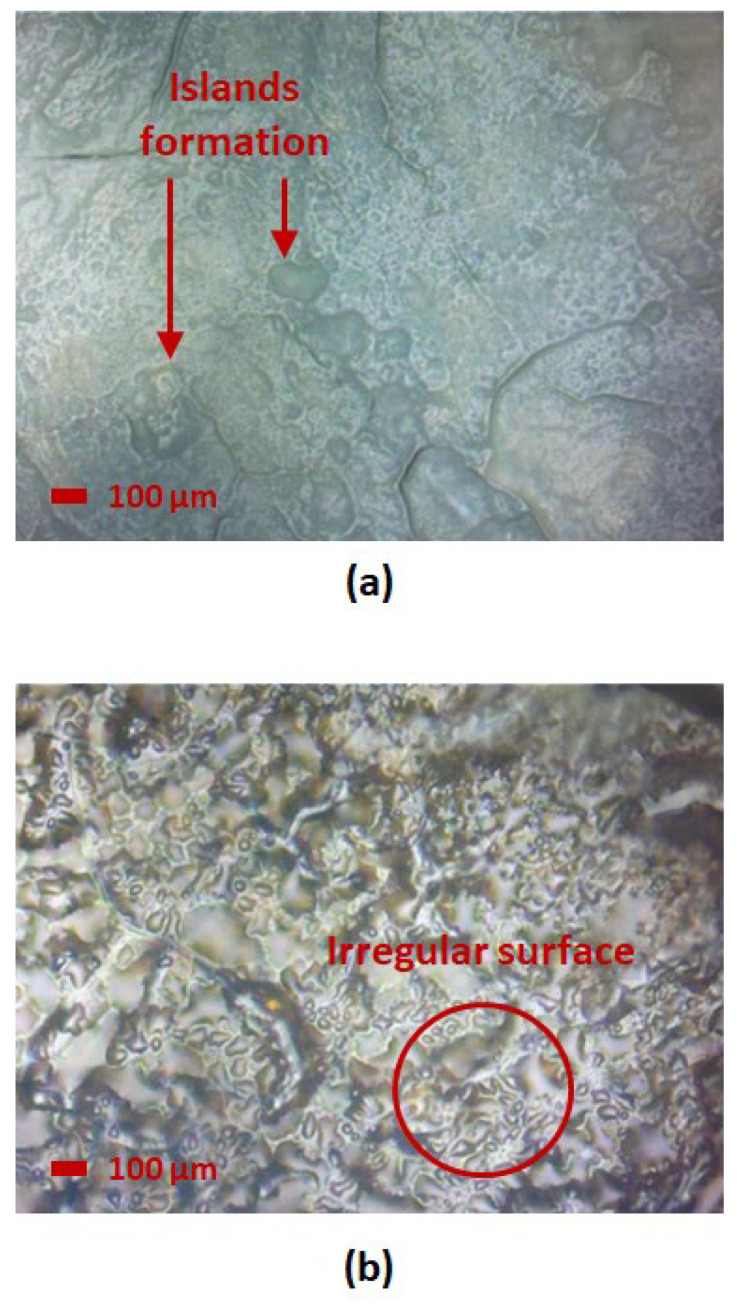
Microscope image at 20× magnification of (**a**) 100 °C and (**b**) 180 °C annealing temperature.

For comparison of annealing temperatures as illustrated in Figure 12, a lower annealing temperature at 100 °C potentially led to incomplete crystallization, where a large number of island structures were distributed randomly over the material surface. Whereas higher annealing temperature at 180 °C led to the formation of an irregular surface with missing pores, potentially due to agglomeration, leading towards the minimal mass change over increasing and decreasing RH as observed from the hygroscopicity test.

This result indicates that optimal annealing conditions enhance the formation of a porous structure, thereby improving the water adsorption and desorption mechanism. On the contrary, samples with under-annealing or over-annealing conditions exhibited minimal porous structures and irregular surfaces, either due to poor crystallinity or agglomeration, which led to high hysteresis or minimal water uptake, as observed in the hygroscopicity test.

**Figure 13:**
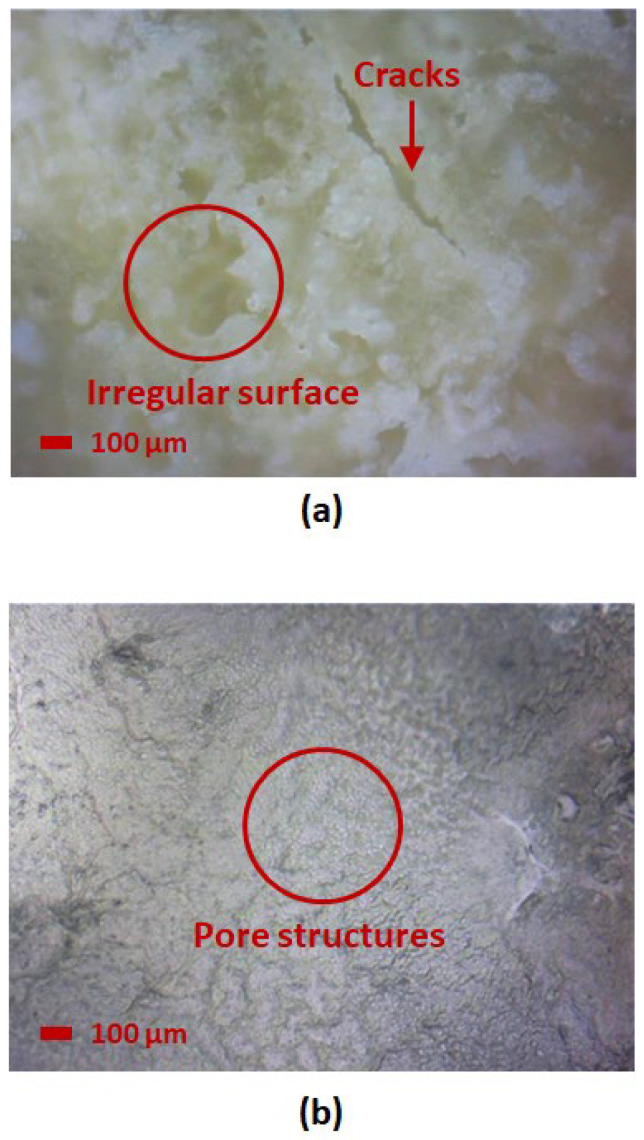
Microscope image at 20× magnification of (**a**) 3 layers SnO_2_-HMT and (**b**) hybrid layers ZnO/SnO_2_-HMT.

Comparing the multilayer and hybrid layer coating in Figure 13, an uneven surface was observed from the samples of 3 layers of SnO_2_-HMT, where the increasing number of coating layers can potentially lead to an uneven crystal structure. Formation of microcracks was also observed in this sample, which can lead to high water retention and low water desorption, resulting in high hysteresis observed from the hygroscopicity test. Whereas for the hybrid layer ZnO/ SnO_2_-HMT, a similar nanostructure with single-layer SnO_2_-HMT with 3 h 140 °C annealing condition was observed, indicating minimal impact of ZnO interlayer on the top surface of SnO_2_-HMT.

### 3.4. Field Emission Scanning Electron Microscope (FESEM)

**Figure 14:**
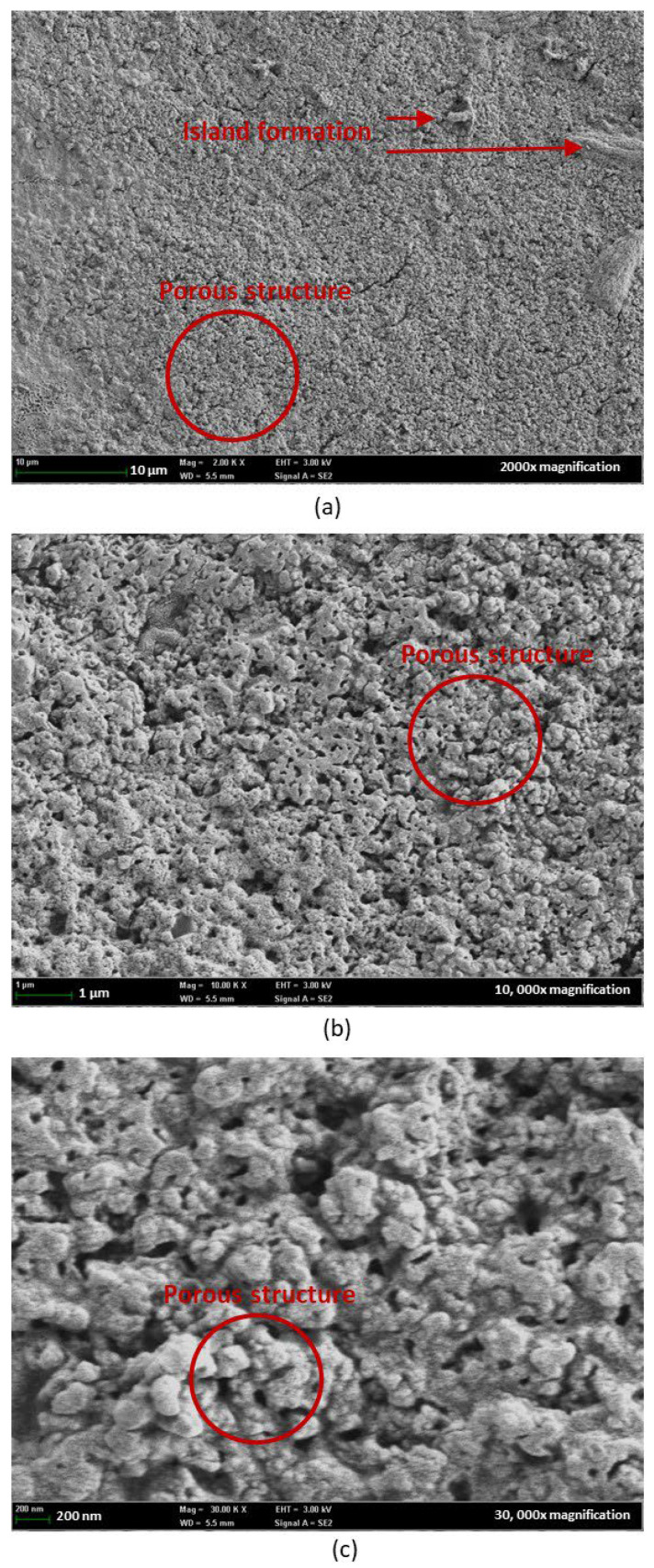
FESEM of SnO_2_-HMT single layer under magnification of (**a**) 2000×, (**b**) 10,000x, (**c**) 30,000×.

**Figure 15:**
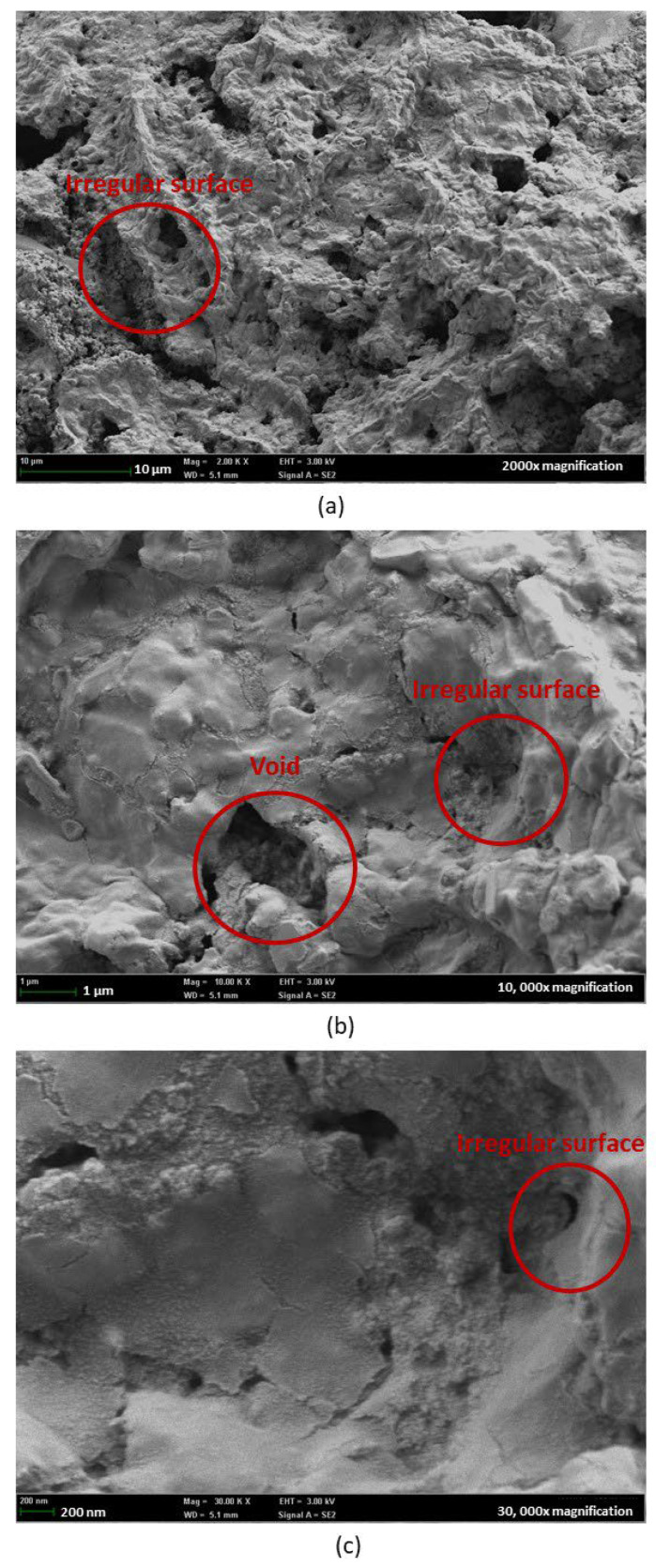
FESEM of SnO_2_-HMT multilayer under magnification of (**a**) 2000×, (**b**) 10,000×, (**c**) 30,000×.

**Figure 16:**
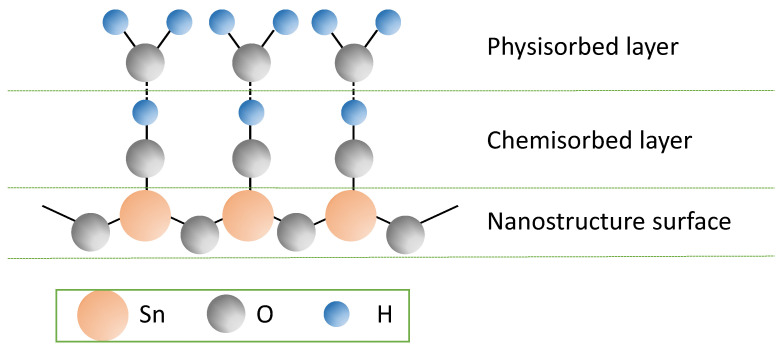
Conceptual schematic diagram of water–surface interaction. Adapted from [14].

FESEM images under 2000×, 10,000×, and 30,000× magnification were captured for the single-layer sample in Figure 14 and the multilayer sample in Figure 15, to further validate the results obtained. The single-layer samples showed minor island formation under 2000× magnification. By increasing the magnification to 10,000× and 30,000×, the porous structures can be clearly observed. These uniform porous structures, which served as active sites for water adsorption and desorption, contribute to the high water–surface interaction physically and chemically with the nanostructure, as shown in Figure 16. High porosity nanostructure also led to a change in surface tension through the capillary effect [33] minimizing the hysteresis of the tested sample.

This explained the observation from the hygroscopicity test, where the uniform porous structures of SnO_2_-HMT annealed under 140 °C for 3 h exhibited minor or negligible hysteresis. On the contrary, increasing the coating layers led to uneven crystal structure and formation of voids, which can be the reason for high hysteresis, as the water molecules might be trapped within the uneven surface or voids, causing poor water desorption [34].

### 3.5. Optical Spectrum Analysis

The FBG sensor was coated with a single layer of SnO_2_-HMT and subjected to the annealing process with optimal annealing conditions, which are 140 °C annealing temperature and 3 h annealing time. To analyze the wavelength shift, the coated FBG was placed in a humidity-controlled chamber and connected to the optical spectrum analyzer (OSA) and light source. The reflected wavelength of the coated FBG was recorded by the OSA at every 10%RH from 40%RH to 80%RH.

**Figure 17:**
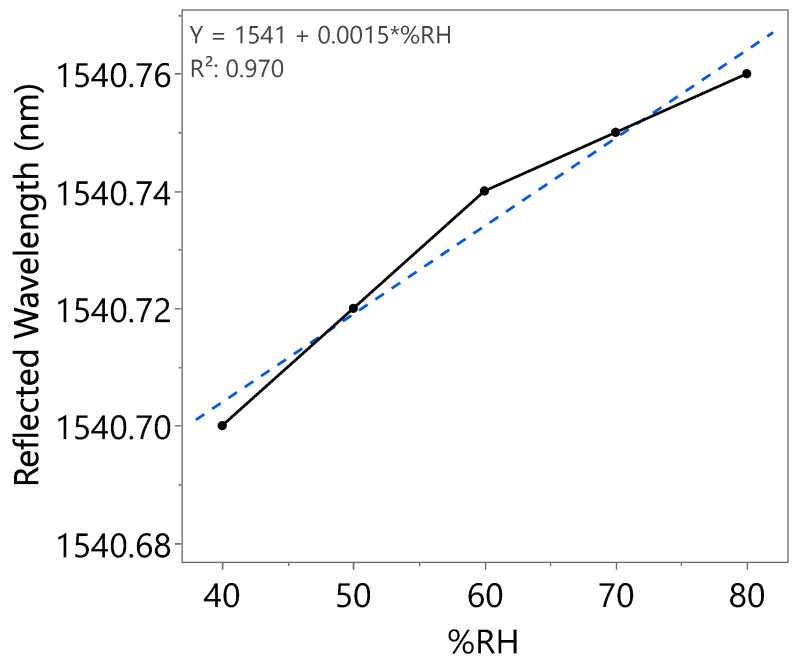
Reflected wavelength of SnO_2_-HMT-coated FBG at different RH levels (first incremental RH cycle).

**Figure 18:**
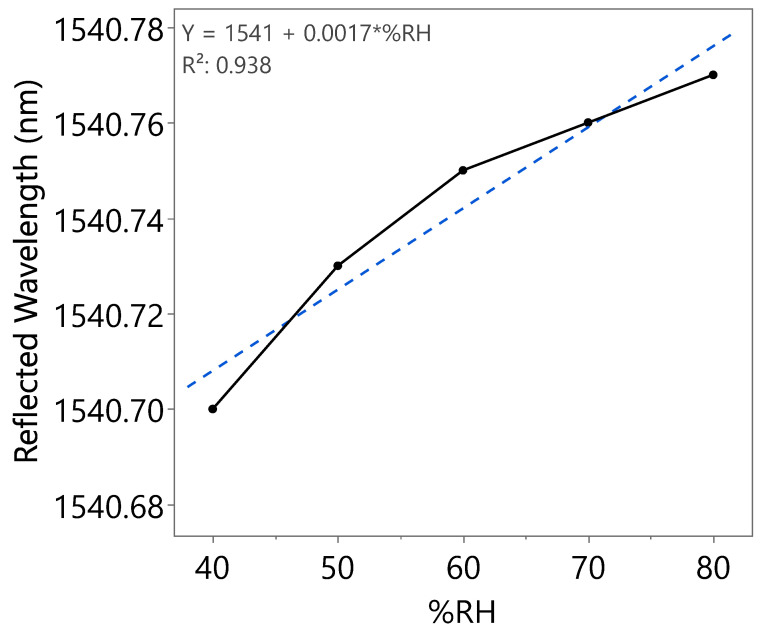
Reflected wavelength of SnO_2_-HMT-coated FBG at different RH levels (second incremental RH cycle).

**Figure 19:**
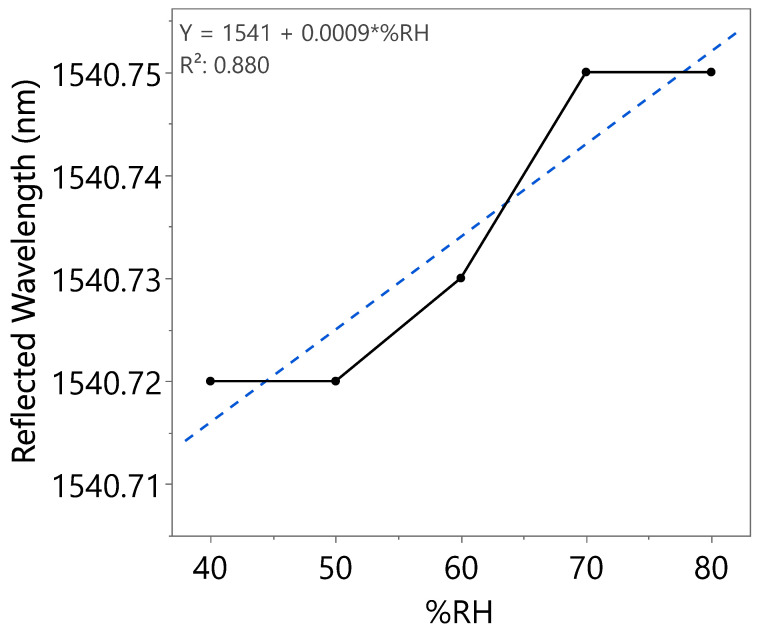
Reflected wavelength of SnO_2_-HMT-coated FBG at different RH levels (third incremental RH cycle).

A wavelength shift with increasing RH can be clearly observed from Figure 17, indicating the effect of coated SnO_2_-HMT towards optical-based humidity sensing, with a sensitivity of 1.5 pm/%RH recorded from 40 to 80%RH in the first incremental RH cycle. Similar works reported a sensitivity of 0.05 pm/%RH from RH ranging from 50 to 80% by coating FBG with ZnO-HMT [35] and sensitivity of approximately 1.5 pm/%RH in the range of 11–83%RH by coating FBG with polyimide [36]. Repeatability tests have been carried out on the same coated FBG as illustrated in Figure 18 and Figure 19. The wavelength shifts were summarized under Table 3. The second incremental RH cycle demonstrated a slight increase in sensitivity to 1.7 pm/%RH. However, a decrease in sensitivity to 0.9 pm/%RH in the third incremental RH cycle was observed. This could be caused by water molecules trapped within the porous structure after exposure to repeated humidity cycles.

## 4. Conclusions

Comparative studies have been conducted in this study to examine the impact of annealing conditions on morphology and structural change in additive-enhanced SnO_2_. By optimizing the annealing time and temperature, the crystallinity can be enhanced through the presence of the HMT additive, resulting in high porosity nanostructures with an increased mass uptake ratio and reduced hysteresis. Over-annealing can lead to decomposition of the additive, suggested by the missing FTIR peak from the range of ~820 cm^−1^ to 1670 cm^−1^, hindering the water adsorption and desorption. Whereas insufficient annealing may result in incomplete crystallization, reducing the effect of the additive in the formation of pore structures. The microscope images have further validated the observation from the hygroscopicity test findings, where samples under over-annealing conditions induced cracks or agglomeration, whereas samples under insufficient annealing conditions were observed to have poor crystallinity.

The study also investigated the increase in coating layers under optimal annealing conditions. The multilayer SnO_2_-HMT samples showed an uneven crystal structure from the microscope images and void formation from FESEM, which traps the water molecules upon adsorption. These findings validate the result from the hygroscopicity test, where the multilayer samples exhibited increased water uptake during increasing RH, but poor water desorption during decreasing RH. The hybrid layer ZnO/SnO_2_-HMT sample showed comparable hygroscopicity and hysteresis to the single-layer SnO_2_-HMT with optimized annealing conditions; this indicates that using ZnO as an interlayer did not enhance the hygroscopicity of the coated nanostructure. Further investigation can be conducted to determine the suitable candidates to act as interlayers of the coated nanostructures.

Overall, the single-layer SnO_2_-HMT or hybrid-layer ZnO/SnO_2_-HMT annealed under 140 °C for 3 h showed the best hygroscopicity among all other samples in this study, with *m value* approximately 0.16 and negligible hysteresis. Sensitivity of 1.5 pm/%RH can be achieved with single-layer SnO_2_-HMT under optimized annealing and coating conditions during the first RH cycle. However, the sensitivity decreased to 0.9 pm/%RH in the third RH cycle during the repeatability test. This result demonstrated that the optimized SnO_2_-HMT can be applied for nanostructure coating on humidity sensors, including optical-based sensing for enhanced sensitivity.

Pure metal oxide nanostructures often exhibit advantages, including high stability, improved optical sensitivity, and large surface area [2]. However, challenges such as self-agglomeration and inconsistent crystallization limited the scalability of the nanostructures for large-scale industry production [37]. Further research, including the development of nanocomposites, interlayer coating for surface modification, and enhanced coating techniques to improve uniformity, is necessary for scalability, reproducibility, and repeatability improvements. This will enable broad applications of the sensor across various humidity-sensing industries, such as civil engineering, agriculture, and semiconductor manufacturing [17,38].

## Figures and Tables

**Table 1:** Metal oxide in various sensing applications.

Metal Oxide	Target Applications	Advantages	Ref
TiO_2_	Chemical/gas sensing	Chemically and thermally stable	[4]
ZnO	Humidity sensing	Chemically and thermally stable	[9]
ZnO/SnO_2_ composite	Humidity sensing	Improved surface area, increased oxygen vacancies	[5]
CeO_2_	Biosensing	Mechanically stable, fast electron transfer kinetics	[10]
Ni-doped Fe_2_O_3_	Biosensing	Non-toxicity, anti-interference	[11]
Cr_2_O_3_	Gas sensing	Corrosion resistant	[12]
V_2_O_5_	Gas sensing	Chemically and thermally stable	[13]
SiO_2_	Humidity sensing	Porous nature	[14]
TiO_2_/ZnO composite	Humidity sensing	Increased oxygen vacancies, good linearity	[15]
SnO_2_-HMT	Humidity sensing	Enhanced hygroscopicity, negligible hysteresis	This work

**Table 2:** Summary of hygroscopicity test performance.

Annealing Time (hours)	Annealing Temperature (°C)	*m value* (Incremental RH)	*m value* (Decremental RH)	Hysteresis
2.5	140	0.1633	0.1807	Slightly higher
3.0	140	0.1644	0.1648	Negligible
3.5	140	0.1632	0.2033	Significant
3.0	100	0.1917	0.1981	Significant
3.0	180	0.0767	0.0533	Slightly higher

**Table 3:** Optical spectrum analysis of three humidity cycles.

Humidity Cycle	%RH	Reflected Wavelength (nm)	Bragg Wavelength Shift (pm)	Sensitivity (pm)
First	40	1540.70	0	1.5
	50	1540.72	20	
	60	1540.74	40	
	70	1540.75	50	
	80	1540.76	60	
Second	40	1540.70	0	1.7
	50	1540.73	30	
	60	1540.75	50	
	70	1540.76	60	
	80	1540.77	70	
Third	40	1540.72	0	0.9
	50	1540.72	0	
	60	1540.73	10	
	70	1540.75	30	
	80	1540.75	30	

## Data Availability

Data is contained within the article.
